# A Case of Thrombocytopenia, Anasarca, Fever, Reticulin Fibrosis/Renal Dysfunction, and Organomegaly (TAFRO) Syndrome Refractory to Treatment With Combination Therapy of Corticosteroid, Tocilizumab, and Rituximab: A Case Report

**DOI:** 10.7759/cureus.110337

**Published:** 2026-06-06

**Authors:** Akihiro Ogawa, Kaori Yamaguchi

**Affiliations:** 1 Division of Nephrology and Rheumatology, Toyama Prefectural Central Hospital, Toyama, JPN

**Keywords:** acute renal failure, corticosteroid, rituximab, tafro syndrome, tocilizumab

## Abstract

We report a case of TAFRO (thrombocytopenia, anasarca, fever, reticulin fibrosis/renal dysfunction, and organomegaly) syndrome in a previously healthy 76-year-old man who was treated with glucocorticoids, tocilizumab, and rituximab. Despite this combination therapy, he developed multiple infections, and the disease progressed, ultimately leading to death.

The patient initially presented with bilateral lower extremity edema and was referred to our hospital for evaluation of severe renal dysfunction and elevated inflammatory markers. Computed tomography revealed mild generalized lymphadenopathy. A bone marrow biopsy showed mild reticulin fibrosis, and a cervical lymph node biopsy demonstrated atrophic germinal centers with prominent interfollicular vascularity, features consistent with Castleman disease-like pathology. Based on these findings, he was diagnosed with TAFRO syndrome. Immunosuppressive treatment was initiated; however, renal dysfunction did not improve, and hemodialysis was initiated three times per week. In addition, infectious complications hindered continuation of therapy. The disease rapidly worsened, and the patient died on day 59 of hospitalization. This case suggests that, in TAFRO syndrome, it is crucial to balance the intensity of immunosuppression with the risk of infectious complications.

## Introduction

TAFRO syndrome is a rare and severe systemic inflammatory disorder first described in Japan in 2010. It is characterized by thrombocytopenia, anasarca, fever, reticulin fibrosis or renal dysfunction, and organomegaly [1]. TAFRO syndrome is often considered a subtype of idiopathic multicentric Castleman disease (iMCD) [2]; however, its distinct clinical features suggest that it may represent a separate disease entity [1].

Although the pathogenesis of TAFRO syndrome remains unclear, dysregulated immune activation and hypercytokinemia are thought to play important roles. Given its potential for rapid clinical deterioration, early diagnosis and prompt treatment are essential. Glucocorticoids are commonly used as first-line therapy, while immunosuppressive agents such as tocilizumab and rituximab are often employed in refractory cases. However, treatment responses are variable, and an optimal therapeutic strategy has not yet been established.

The prognosis for TAFRO syndrome remains poor due to challenges in diagnosis, low disease recognition, and the lack of established treatments. The five-year survival rate has been reported to be approximately 66.5%, with a marked decline in overall survival after 24 months of disease progression, with more than one-third of patients dying [3,4].

Here, we report a refractory case of TAFRO syndrome requiring hemodialysis despite combination therapy with glucocorticoids, tocilizumab, and rituximab. This case highlights the challenges in disease management and provides insight into the heterogeneity of treatment responses in TAFRO syndrome.

## Case presentation

**Figure 1:**
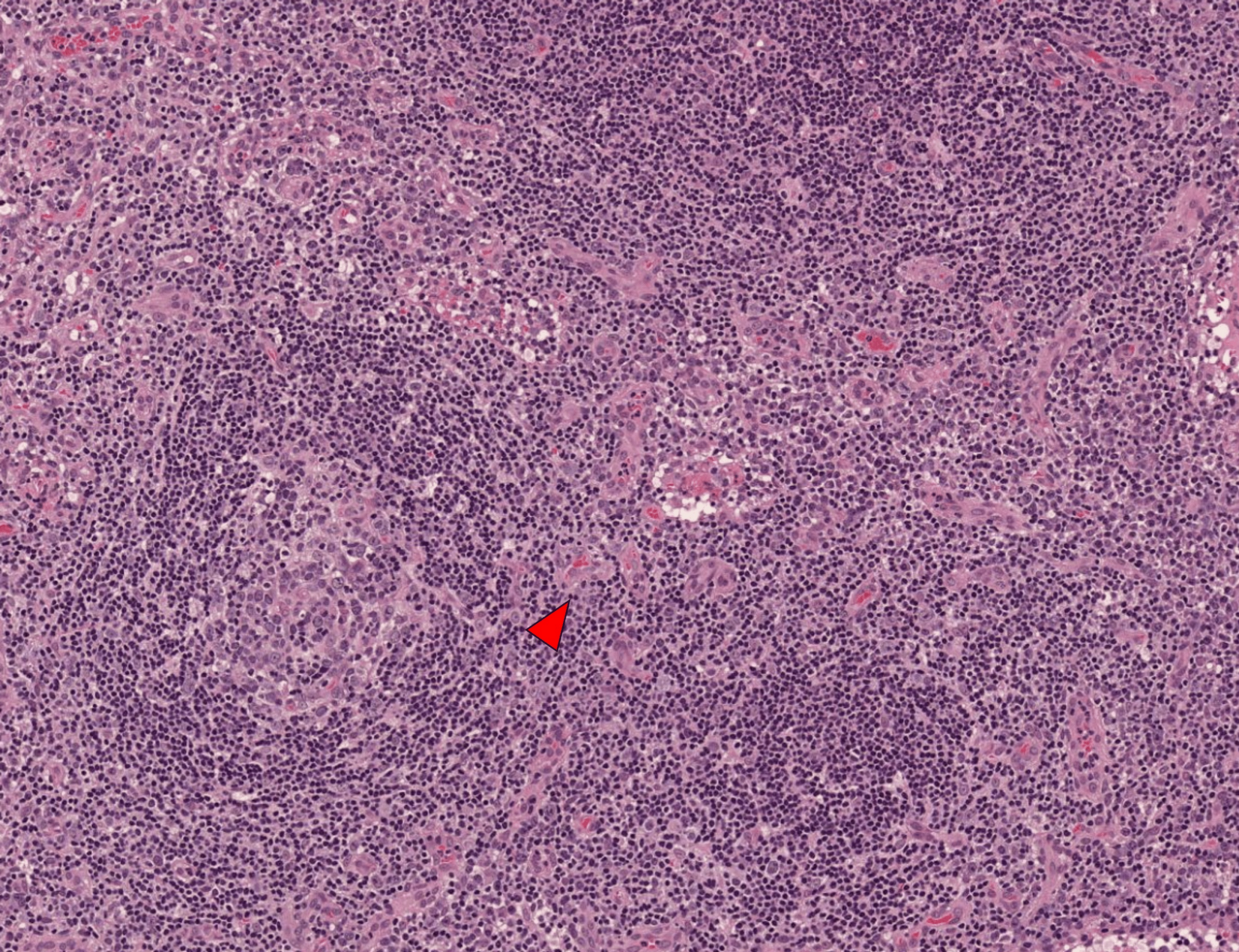
Lymph node biopsy showing atrophic germinal centers with prominent interfollicular vascularity (red arrowhead).

**Figure 2:**
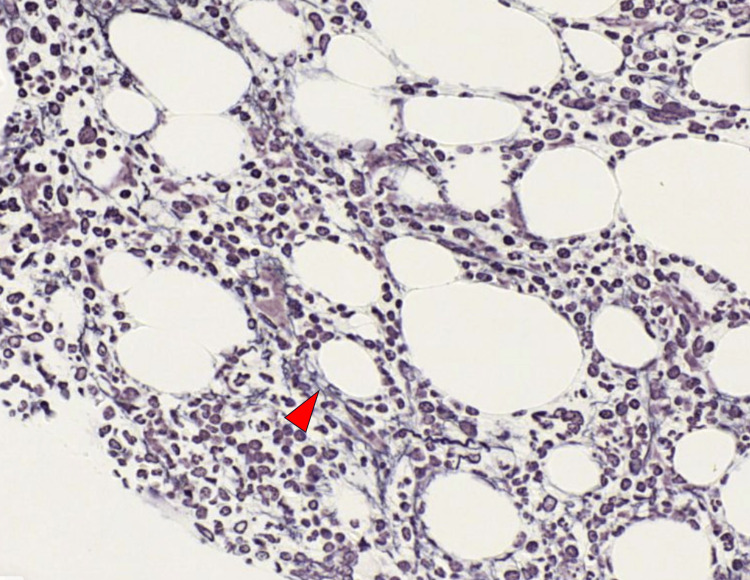
Bone marrow examination (sliver impregnation) showing mild increased reticulin fibrosis (red arrowhead).

A 76-year-old previously healthy man presented to a local clinic with a one-month history of general fatigue. A cervical lymph node biopsy was performed on hospital day three, followed by a bone marrow biopsy on day 4. Lymph node biopsy demonstrated Castleman disease-like features, including atrophic germinal centers and interfollicular vascular proliferation (Figure 1). Bone marrow biopsy revealed mild reticulin fibrosis (Figure 2).

Based on these clinical and histopathological findings, the patient was diagnosed with TAFRO syndrome. Hence, steroid pulse therapy (intravenous methylprednisolone 500 mg/day for three days) with subsequent oral steroid therapy (prednisolone 40 mg/day) was started. 

Despite diuretic therapy, because of worsening renal function, decreased urine volume, and volume overload, hemodialysis was initiated on day 8 via a dialysis catheter. Then renal function did not improve, and he switched to maintenance dialysis three times a week. Tocilizumab at a dose of 8 mg/kg was initiated on day 13 because of elevated IL-6 levels and persistent systemic inflammation, residual pleural effusion and ascites, and poor response to steroid therapy. However, no improvement in renal function or fluid retention was observed. Subsequently, a total of 2 cycles of rituximab (375 mg/m², weekly) were administered.

On hospital day 29, the patient developed a CMV infection, and ganciclovir treatment was initiated, and prednisolone was tapered off. On hospital day 40, the patient developed septic shock secondary to a catheter-associated bloodstream infection caused by Enterococcus faecium, requiring vancomycin (0.5 g/day) treatment and admission to the intensive care unit (ICU). Empirical antimicrobial therapy with tazobactam/piperacillin was initiated, continuous intravenous noradrenaline and vasopressin were administered to maintain hemodynamic stability, and continuous hemodiafiltration (CHDF) was initiated.

**Figure 3:**
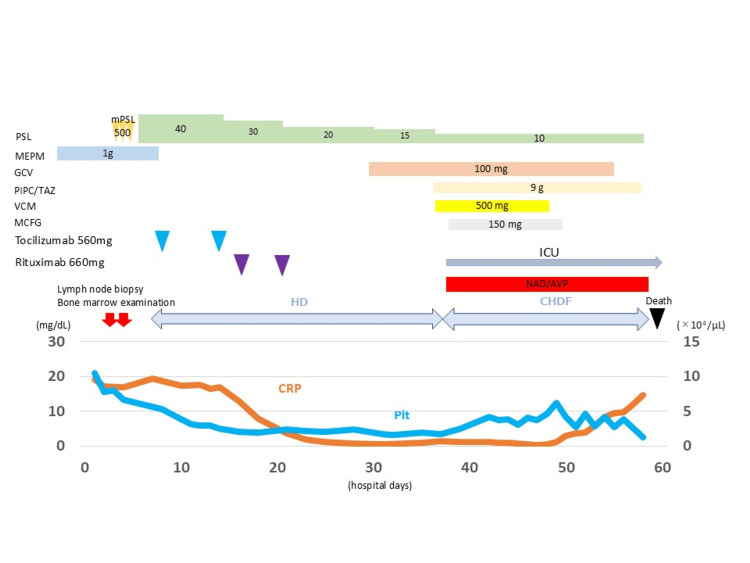
Clinical course of the patient. PSL: prednisolone; mPSL: methylprednisolone; MEPM: meropenem; GCV: ganciclovir; PIPC/TAZ: tazobactam/piperacillin; VCM: vancomycin; MCFG: micafungin; NAD: noradrenaline; AVP: arginine vasopressin; CRP: C-reactive protein; Plt: platelet; HD: hemodialysis; CHDF: continuous hemodiafiltration; ICU: intensive care unit. The figure was created using Microsoft PowerPoint.

Two days later, *Candida* was detected in the blood culture, and treatment with micafungin was initiated. The detailed diagnosis and treatment timeline is illustrated in Figure 3.

The blood CMV-DNA levels decreased, and the blood cultures also became negative. Although antimicrobial therapy achieved partial control of the infections, CRP levels continued to rise, and pleural and peritoneal effusions worsened. Worsening disease activity of TAFRO syndrome was suspected, but further immunosuppressive therapy was deemed contraindicated due to the high risk of infection. The patient’s condition progressively deteriorated, and he died on hospital day 59.

## Discussion

TAFRO syndrome was first reported in Japan in 2010 and is a rare, systemic inflammatory disease characterized by thrombocytopenia, anasarca, fever, reticulin fibrosis or renal dysfunction, and organomegaly [1]. It is sometimes considered a special subtype of idiopathic multicentric Castleman disease (iMCD) because lymph node biopsies often reveal the pathology findings seen in iMCD [2]. In Iwaki‘s criteria, the pathological features of lymph nodes of patients with TAFRO syndrome were defined as atrophy of the germinal center with enlargement of endothelial cell nucleus, proliferation of interfollicular endothelial vein, and rare mature plasma cells [5]. However, other characteristic TAFRO findings, such as a normal immunoglobulin level, thrombocytopenia, relatively small lymphadenopathy, marked pleural effusion, ascites and edema, renal insufficiency, reticulin myelofibrosis, and acute or subacute onset and clinical course, are not considered typical findings for iMCD [1]. Therefore, TAFRO syndrome may be a disease entity distinct from iMCD.

Although the precise etiology remains unclear, secondary hypercytokinemia, triggered by infections, autoimmune processes, or malignancies, has been proposed as a pathogenic mechanism. The central role is played by interleukin-6 (IL-6), which induces activation of the Janus kinase (JAK)-signal transducer and activator of transcription (STAT) signaling pathway via the IL-6 receptor. This activation promotes immune cell activation and further release of proinflammatory cytokines, resulting in the formation of a positive feedback loop of inflammation. In particular, IL-6-induced expression of vascular endothelial growth factor (VEGF) increases vascular permeability, leading to systemic edema as well as pleural and ascitic fluid accumulation. However, in TAFRO syndrome, many cases cannot be fully explained by a single IL-6-dependent mechanism. Instead, the underlying pathology is thought to resemble a cytokine storm involving multiple cytokines acting simultaneously. These cytokines further amplify each other through multiple signaling pathways, ultimately causing excessive activation of the immune system [6].

TAFRO syndrome is diagnosed based on a combination of major and minor criteria. The major criteria include anasarca (pleural effusion, ascites, and/or peripheral edema), thrombocytopenia (platelet count <100,000/μL in the absence of bone marrow suppression), and systemic inflammation (fever >37.5℃ and/or C-reactive protein ≥2 mg/dL). The minor criteria consist of Castleman disease-like histopathological findings in lymph nodes, reticulin fibrosis, and/or megakaryocytic hyperplasia in the bone marrow, mild organomegaly (hepatomegaly, splenomegaly, and/or lymphadenopathy), and progressive renal dysfunction.

A definitive diagnosis requires the presence of all three major criteria and at least two of the four minor criteria. In addition, alternative diagnoses must be carefully excluded, including malignancies such as lymphoma and multiple myeloma, autoimmune disorders, infectious diseases, POEMS syndrome, liver cirrhosis, thrombotic thrombocytopenic purpura (TTP), and hemolytic uremic syndrome (HUS) [1].

In the present case, the patient fulfilled all three major criteria, anasarca, thrombocytopenia, and systemic inflammation, as well as three minor criteria, including Castleman disease-like lymph node histopathology, reticulin fibrosis in the bone marrow, and progressive renal dysfunction. Based on these findings, a diagnosis of TAFRO syndrome was established. The calculated severity score corresponded to Grade 4. Comprehensive evaluation revealed no evidence of infection, autoimmune disease, malignancy, liver cirrhosis, thrombotic thrombocytopenic purpura, or hemolytic uremic syndrome. These findings further supported the diagnosis of TAFRO syndrome.

Patients with TAFRO syndrome require accurate diagnosis and prompt initiation of treatment due to the potential for rapid systemic deterioration, but no standardized evidence-based treatment regimen has been established. Most patients with TAFRO syndrome are treated initially with corticosteroids, exerting anti-inflammatory effects through inhibition of multiple cytokines and inflammatory mediators. However, in some cases, steroid monotherapy may not be effective [7]. In previous studies, high hemoglobin and/or low CRP levels were predictors of response to steroid monotherapy [5]. In this case, the patient had a high level of systemic inflammation, and we initiated tocilizumab, second-line immunosuppressive agents.

Tocilizumab, a humanized monoclonal antibody against the IL-6 receptor, is commonly employed due to the suspected central role of IL-6 in TAFRO syndrome. Nevertheless, not all patients exhibit markedly elevated IL-6 levels, suggesting heterogeneity in pathogenesis. In such patients, the clinical benefit of IL-6 inhibition may be attenuated. Iwaki et al. reported that there was little evidence of an association between the serum IL-6 concentration and effectiveness of anti-IL-6 therapy with tocilizumab in TAFRO syndrome, suggesting that elevated IL-6 may not be the primary pathological driver of the proinflammatory hypercytokinemia responsible for TAFRO syndrome [5].

In our case, a temporary stabilization of the disease raises the possibility because progression of pleural and ascitic fluid accumulation was suppressed, and there was no worsening of CRP levels following tocilizumab administration. Disease exacerbation coincided with presumed waning of tocilizumab activity, implying that ongoing IL-6 inhibition may have contributed to partial disease control. However, corticosteroid therapy had minimal effect, and subsequent administration of tocilizumab also failed to achieve sustained disease control.

Additionally, while plasma cell proliferation is rare in TAFRO syndrome, in certain cases, plasma cells infiltrate the interfollicular zone of lymph nodes, with IL-6 facilitating B-cell maturation and enhancing immunoglobulin production. It is speculated that in such cases, RTX could prove useful as a main component of the therapeutic regimen; we initiated Rituximab, third-line immunosuppressive agents. Although B-cell depletion was confirmed following rituximab, there was no associated clinical improvement, suggesting limited therapeutic impact.

Rituximab targets CD20-positive B cells but does not eliminate CD20-negative plasma cells, which may continue to produce pro-inflammatory cytokines, including IL-6. Persistent cytokine production from these cells could sustain systemic inflammation despite successful B-cell depletion. This mechanism may partly explain the limited efficacy of rituximab observed in the present case.

The clinical course was further complicated by the development of multiple opportunistic infections, including cytomegalovirus viremia, candidemia, and catheter-related bloodstream infection. These complications restricted the use of further immunosuppressive therapy and are thought to have contributed to the patient’s deterioration, as immunosuppressive agents had to be reduced.

Although the patient ultimately had a fatal outcome, it remains possible that earlier recognition of disease progression and more rapid escalation of therapy may have influenced the clinical course. In particular, earlier introduction of additional immunosuppressive agents such as calcineurin inhibitors or rituximab, as well as closer reassessment of response to tocilizumab, might have provided an opportunity for improved disease control before the onset of severe opportunistic infections. However, the optimal timing and sequencing of these therapies in refractory TAFRO syndrome remain unclear.

One of the key challenges in the treatment of TAFRO syndrome is how to balance adequate immunosuppression with the risk of severe infections. Patients with TAFRO syndrome often present with a highly inflammatory state, necessitating prompt and intensive immunosuppressive therapy; however, excessive immunosuppression increases the risk of opportunistic infections, which can be life-threatening. Treatment for this syndrome includes corticosteroids, IL-6 inhibitors, and rituximab, which are often used in combination. While these therapies are effective in controlling inflammation, they may also impair host defense mechanisms through suppression of cellular immunity and B-cell depletion. Therefore, careful monitoring for the development of infections during treatment is essential. In addition, treatment intensity should be dynamically adjusted according to disease activity and infection risk to achieve both rapid control of inflammation and minimization of adverse events. In the present case, multiple infections occurred as a result of intensive immunosuppressive therapy, necessitating treatment modification. This suggests that individualized risk assessment and rigorous clinical monitoring are crucial in the management of TAFRO syndrome.

In conclusion, this case highlights the therapeutic challenges posed by TAFRO syndrome. Although corticosteroids, tocilizumab, and rituximab offered limited and partial benefits, none were sufficient to halt disease progression. The heterogeneous clinical manifestations and variable treatment responses in TAFRO syndrome underscore the need for individualized, pathophysiology-driven treatment approaches. Further research and accumulation of clinical data are essential to establish optimal management strategies for this complex and potentially fatal disorder.

## Conclusions

We encountered a severe and rapidly progressive case of TAFRO syndrome complicated by acute renal failure, thrombocytopenia, and systemic inflammation, which proved refractory to treatment with corticosteroids, tocilizumab, and rituximab. Despite aggressive immunosuppressive therapy, the patient’s condition deteriorated and resulted in death. This case highlights the critical importance of early recognition and prompt therapeutic intervention in TAFRO syndrome. Given the disease’s heterogeneous clinical presentations and variable responses to treatment, future investigations to further elucidate the pathophysiology of TAFRO syndrome, together with continued accumulation of clinical cases and prospective studies, may help establish more effective and safer treatment strategies for this challenging disease.
